# Dr. M. T. Irby, Corinth, Mississippi

**Published:** 1873-09

**Authors:** 


*Dr. M. T. Irby, Corinth, Mississippi.*

The formula you refer to was "set up" by the printer from "copy," just as you see it. The hyeroglyphics you mention were doubtless intended for lbs. We know nothing further of the formula.

